# Knowledge, attitude, practice and associated factors about voluntary blood donation among regular undergraduate students of Wachemo University, Southcentral Ethiopia: a cross-sectional study

**DOI:** 10.3389/fpubh.2024.1485864

**Published:** 2024-11-19

**Authors:** Abdulhakim Mussema, Bethelhem Nigussie, Biruk Anmaw, Habtamu Abera, Habib Nageso, Solomon Gebre Bawore, Amina Shemsu, Dagmawi Woldesenbet, Kemal Mohammed, Abdurezak Mohammed Seid, Dawit Admasu

**Affiliations:** ^1^Department of Medical Laboratory Science, Wachemo University, Hosanna, Ethiopia; ^2^Public Hospital, Addis Ababa, Ethiopia; ^3^Negele Arsi General Hospital and Medical College, Arsi Negele, Ethiopia; ^4^Department of Nursing, Wachemo University, Hosanna, Ethiopia

**Keywords:** attitude, Ethiopia, knowledge, practice, voluntary blood donation

## Abstract

**Introduction:**

Human blood plays a crucial role in transporting metabolic waste and essential minerals, including oxygen, to cells. Blood transfusions are a critical intervention in various situations such as trauma, inherited bleeding disorders, childbirth, and numerous medical and surgical procedures, often being the only means to prevent death. A significant challenge, particularly in developing nations, is maintaining a sufficient supply of safe blood. An individual’s mindset, beliefs, and familiarity with blood donation significantly affect their willingness to donate. This research aimed to evaluate the knowledge, attitudes, practices, and related factors concerning voluntary blood donation among undergraduate regular students at Wachemo University in Southcentral Ethiopia.

**Materials and methods:**

A cross-sectional study was conducted from October 19 to November 10, 2023, using a stratified random sampling method to select participants. Data were gathered through self-administered structured and semi-structured questionnaires. The responses regarding knowledge, attitudes, and practices were analyzed using SPSS version 24 and presented through text, figures, and tables.

**Result:**

A total of 393 respondents participated in the study (97.76% response rate), comprising 59% males and 41% females. Of the participants, 77.6% demonstrated adequate knowledge, 79.6% exhibited positive attitudes, while only 19.3% had engaged in blood donation. Factors influencing these outcomes included knowledge, previous residence, and college background. Reluctance to donate blood was primarily linked to fear and concerns about time constraint.

**Conclusion and recommendations:**

The findings indicate that while a majority of students possess good knowledge (77.6%) and favorable attitudes (79.6%) toward blood donation, only a small fraction (19.3%) actively participates in the practice. It is recommended that the institution collaborate with relevant stakeholders to address the factors affecting voluntary blood donation among students. In addition, incorporating topics on blood donation and its significance into the university curriculum can foster a culture of generosity among students. This strategy is essential for improving blood donation rates in this region.

## Introduction

Blood transfusion is a vital aspect of contemporary healthcare, significantly contributing to the saving of millions of lives annually (1). It serves as a critical intervention not only in emergencies, such as trauma or surgical procedures, but also in managing chronic conditions like anemia, cancer, and various blood disorders (2). The ability to conduct increasingly advanced medical and surgical procedures is heavily dependent on the availability of safe blood, which greatly improves both life expectancy and quality of life for patients dealing with acute and chronic health issues (3).

Timely access to safe blood is crucial for all healthcare facilities. However, many developing countries encounter significant obstacles, resulting in a widening gap between the demand for blood and its availability (4, 5). The capacity of a healthcare system and its coverage of the population are key factors in determining a country’s blood needs. In developed nations, the demand for blood continues to rise due to advancements in medical technology and the complexity of surgical procedures, highlighting the need for a dependable blood donation system to support healthcare efforts (6).

The World Health Organization (WHO) promotes 100% voluntary blood donation to ensure a stable and safe blood supply (3). Despite this recommendation, underdeveloped countries—home to over 80% of the global population—often depend on replacement or paid donors for around 60% of their blood supply (7, 8). This reliance raises ethical issues and concerns about the safety and reliability of donated blood (6, 8). Ethiopia, in particular, has the lowest percentage of voluntary blood donors among WHO’s African member states, currently at a troubling 22% (9). This statistic underscores the urgent need for targeted initiatives to encourage voluntary blood donation.

The implications of an insufficient blood supply are severe, especially in developing nations like Ethiopia (10). A staggering statistic indicates that 99% of the 500,000 women who die each year during pregnancy and childbirth reside in these countries, often due to complications such as hemorrhage that require blood transfusions (11). Hemorrhage is the leading cause of maternal mortality worldwide (11). Currently, the National Blood Bank Service of Ethiopia provides safe blood and blood products to only 52% of medical facilities, creating a significant gap in maternal healthcare services (12). Research across sub-Saharan Africa, including Ethiopia, has shown a strong link between maternal mortality rates and the inability to access necessary blood transfusions (13–15). Notably, maternal hemorrhage accounts for 26% (with variations from 16 to 72%) of all maternal deaths (16).

Ethiopia has an estimated population exceeding 120 million, making it the second most populous country in Africa. The nation faces considerable public health challenges, ranking among the top 10 globally for motor vehicle accidents and experiencing high rates of non-immune malaria cases. Consequently, many medical treatments require a consistent supply of blood; indeed, one in seven hospital patients will need a transfusion at some point during their care (8).

In line with WHO standards, Ethiopia collected only 223,000 units of blood during the 2019–2020 period—just 22% of what is necessary to meet its healthcare needs (16, 17). Currently, there have been few studies examining the levels of knowledge, attitudes, and practices related to blood donation in various regions of Ethiopia (10, 18–21). Several factors contribute to this shortfall: limited opportunities for donation, busy academic schedules among potential donors, widespread ignorance about the importance and process of blood donation, fear of needles or infection, and pervasive misconceptions regarding the effects of blood donation on personal health. Addressing these issues is crucial for cultivating a culture of voluntary blood donation among students and the broader community (8, 16).

Blood donation is a vital component of healthcare systems, providing necessary support for various medical treatments and emergencies. In Ethiopia, however, voluntary blood donation remains critically low, particularly among the youth. Understanding the mechanisms of knowledge, attitude, and practice (KAP) surrounding blood donation among undergraduate students is essential for fostering a culture of voluntary giving.

Knowledge is a fundamental component influencing attitudes toward blood donation. Studies have shown that individuals who possess accurate information about the safety and benefits of blood donation are more likely to develop positive attitudes toward donating (22). For instance, misconceptions about health risks and eligibility criteria can deter potential donors. In the context of Wachemo University, where students are often future healthcare professionals, their understanding of blood donation’s importance could significantly impact their willingness to donate. Attitude is another crucial determinant in the KAP framework. Positive attitudes toward blood donation can lead to increased likelihood of participation; however, negative perceptions often rooted in cultural beliefs or lack of awareness can create barriers (23). For example, if students view blood donation as a risky or painful procedure, they may be less inclined to participate. Therefore, assessing students’ attitudes toward blood donation is essential for identifying potential barriers and facilitators.

Furthermore, the practice of blood donation is often influenced by both knowledge and attitude. A study by Eltewacy et al. (24) found that while students may express positive attitudes toward donating blood, a lack of practical experience or understanding of the donation process can impede actual participation. This gap between intention and action highlights the need for targeted educational interventions that not only inform but also engage students in the donation process.

At Wachemo University, understanding the knowledge, attitudes, and practices (KAP) of regular undergraduate students regarding blood donation is crucial. The university’s diverse student population presents a unique opportunity to investigate the factors influencing blood donation behaviors within this demographic. By identifying the barriers that students face, we can establish a strong foundation for educational initiatives aimed at raising awareness about the importance of voluntary blood donation (18, 25).

Given the pressing need for safe blood in Ethiopia and the potential of university students to help meet this demand, this study aims to provide valuable insights into fostering a culture of voluntary blood donation among the youth. The findings will not only enrich academic literature but also guide policymakers and health educators in developing effective strategies to improve blood donation rates nationwide. Promoting a culture of voluntary donation among young people can help bridge the gap between blood supply and demand, ultimately saving lives and improving health outcomes across Ethiopia. This research will assess students’ knowledge levels, attitudes toward blood donation, and actual practices, identifying gaps that can be addressed through targeted educational interventions.

## Materials and methods

### Study area and study period

A cross-sectional study was employed to assess the KAP regarding blood donation among undergraduate students at Wachemo University (WCU) in Hosanna, located in the Hadiya Zone of Central Ethiopia, approximately 230 km from Addis Ababa. WCU spans over 200 hectares and consists of three campuses: the main campus, NEMMCSH campus, and Durame campus. The university currently has an enrollment of over 18,400 students across 48 departments within six colleges: Engineering and Technology, Natural and Computational Sciences, Medicine and Health Sciences, Agricultural Sciences, Business and Economics, and Social Sciences and Humanities.

The research was carried out from October 19 to November 10, 2023, in response to the pressing need to improve voluntary blood donation rates in Ethiopia, particularly among young adults. By utilizing the Knowledge, Attitude, and Practice (KAP) framework, this study aims to identify specific areas where educational interventions can be most effective. Focusing on regular undergraduate students at Wachemo University allows for localized insights that can contribute to addressing existing gaps in the literature and inform broader strategies for enhancing blood donation initiatives.

### Study design

An institutional-based cross-sectional study design was used to assess the knowledge, attitude, and practice of regular students toward voluntary blood donation at WCU. This design was chosen for its efficiency in gathering data from a large sample within a short timeframe, which provides a snapshot of current knowledge and attitude related to blood donation, which can inform targeted interventions. The study utilized a quantitative research approach, allowes for statistical analysis to identify patterns and correlations among the factors influencing voluntary blood donation.

### Study population and eligibility criteria

The source population for this study comprised all regular undergraduate students currently enrolled at Wachemo University. The study population comprised students who were enrolled and attended classes during the data collection period. The inclusion criteria for the study were set to encompass all regular students attending Wachemo University during the specified timeframe and provide informed consent to participate in the study. Conversely, certain exclusion criteria were established to refine the study population further. Additionally, students who were seriously ill and unable to participate were also excluded to ensure that the data collected reflected the perspectives of those who were actively engaged in campus life. This population is particularly relevant for several reasons. First, university students represent a demographic that is often healthy, socially active, and engaged in community service, making them ideal candidates for voluntary blood donation initiatives. Furthermore, students in this age group are typically more open to learning and adopting new behaviors, which can facilitate positive changes in attitudes toward blood donation.

### Sample size determination

The sample size for this study was determined using Cochran’s formula for estimating a single population proportion, represented as (Z(*α*/2))^2^ · p · (1−p)/d^2^ (26). This calculation considered a 95% confidence interval, a 5% margin of error, and a favorable attitude proportion of 47.4% toward voluntary blood donation among regular students at Ambo University (19). In this context, p denotes the population proportion (47.4%), d represents the margin of error (5%), and Z(*α*/2) corresponds to a 95% confidence level, which is 1.96. By substituting these values into the formula, we obtained:

*n* = (1.96)^2^ * 0.474 * (1–0.474)/0.0025 ≈ 383

Considering a 5% non-response rate, the final sample size amounted to 402.

### Sampling method and procedure

A stratified random sampling method was used to ensure representation across various faculties and years of study. This approach help capture diverse perspectives and experiences related to blood donation. The sampling process consisted of three stages. First, four colleges were selected from the total pool of colleges. In the second stage, 19 departments were chosen from these colleges. Finally, specific years of study were identified for sampling.

Students were randomly selected based on their identification numbers. In total, there were 1,292 regular students enrolled in the specified year of study across the four colleges and nineteen departments. After identifying the number of students in each department based on their year of study, the sample was allocated using a proportional allocation formula that reflects the relative size of each department’s student population compared to the total student population across all selected departments. The formula is as follows: *n*ᵢ = (*N*ᵢ/*N*) × *n*.

Where: *n*ᵢ = number of students to be sampled from department i

*N*ᵢ = total number of students in department i

*N* = total number of students across all selected departments

*n* = desired total sample size

Once we calculated the proportional sample size for each chosen department and academic year, we engaged in collaboration with the relevant departments to facilitate student recruitment. During this process, we ensured that students received a comprehensive overview of the study, including its objectives and the requirements for participation. Then participants were selected through simple random sampling techniques.

### Study variable

The dependent variables include knowledge, attitude, and practice of voluntary blood donation. In contrast, the independent variables encompass age, sex, religion, marital status, college, department, academic year, previous residence, and ethnic group.

### Data collection techniques and tools

#### Data collection tools

Data was collected through a structured questionnaire comprising sections on demographic information, knowledge assessment, attitude measurement, and self-reported blood donation practices. Prior to full deployment, the questionnaire underwent pre-testing to ensure clarity and reliability. Its reliability was evaluated using Cronbach’s Alpha, a metric for internal consistency. The Cronbach’s Alpha scores for the knowledge and attitude sections were 0.71 and 0.74, respectively.

It comprised four sections:

- Socio-demographic factors, which included information on age, gender, department, and residence.
- Knowledge questions that assessed students’ understanding of blood donation processes and their significance.
- Attitude questions aimed at evaluating perceptions and feelings toward blood donation.
- Practice questions designed to gather data on past experiences with blood donation and future intentions to donate.

#### Data collection techniques

After randomly selecting identification numbers from each department, a data collector was assigned to each of the chosen students. Before starting the data collection, the purpose of the study was explained to the participants. Special attention was given to clarifying the significance and meaning of each question, ensuring that the data collector could explain them in an understandable manner if needed.

#### Data quality assurance

To ensure data quality, the questionnaire was developed based on existing literature and relevant studies related to blood donation (9, 10, 18–21, 25, 27). This questionnaire was slightly modified to align with the study’s objectives and context. Pretest was performed. During data collection, data collectors performed checks for consistency and completeness of responses before entering the data into computer software for analysis.

#### Data processing and analysis

Descriptive statistics were utilized to summarize the demographic characteristics of the participants, as well as their Knowledge, Attitudes, and Practices (KAP) scores. To investigate the relationships between knowledge, attitudes, and actual blood donation practices, inferential statistics were employed, specifically regression analysis, using SPSS version 24.

For selecting variables in the bivariate logistic regression analysis, a *p*-value threshold of below 0.25 was used. Following this, multivariable logistic regression analyses were performed to investigate the independent associations between the variables identified in the bivariate analysis and the actual practice of VBD. A *p*-value of less than 0.05 at a 95% confidence level in the multivariable logistic regression analysis was deemed to indicate a statistically significant association.

Each question in the knowledge and attitude sections of the questionnaire was scored with 1 point for correct answers and 0 points for incorrect ones. The total score for each participant was determined by summing the points from all applicable questions. A threshold of 50% was established to represent a basic level of understanding or a positive attitude toward blood donation. This cutoff enables differentiation between individuals who possess foundational knowledge or a positive attitude and those who do not. This method is frequently employed in educational assessments and research to indicate competency (10, 21, 27).

Participants who achieved a mean score or higher on the knowledge assessment were categorized as having a good level of knowledge regarding blood donation. Conversely, those who scored below the mean were classified as having a poor knowledge level.

To evaluate attitudes toward blood donation, we posed questions that examined general perceptions and intentions related to future donations. Individuals whose attitude scores met or exceeded the mean were deemed to have a favorable attitude toward blood donation. Those with scores falling below the mean were considered to have an unfavorable attitude.

Additionally, blood donation practice was defined based on individuals’ experiences; specifically, those who had participated in at least one blood donation were classified as having engaged in the practice. This classification allowed us to distinguish between theoretical knowledge and practical involvement in blood donation activities.

### Ethical consideration

Ethical clearance and approval were obtained from the School of Medical Laboratory Science with the reference number of MLS231/2015. Verbal consent was acquired from all study subjects, assuring them that participation was voluntary and that confidentiality would be maintained. To protect participants’ anonymity, code numbers were used instead of personal identifiers, and all completed questionnaires were sealed after data collection in each department.

### Operational definition

#### Knowledge

This refers to the level of understanding students have regarding the benefits, risks, and eligibility criteria for blood donation. Participants who correctly answer 50% or more of the questions designed to assess knowledge are classified as having good knowledge, while those who score below 50% are classified as having poor knowledge (10, 21, 27).

#### Attitude

Attitude is defined as the participants’ intentions toward voluntary blood donation practices. Those who answer 50% or more of the attitude assessment questions positively are categorized as having a favorable attitude, whereas those who score below 50% are deemed to have an unfavorable attitude (10, 21, 27).

#### Practice

Individuals who have participated in blood donation at least once in their lifetime are considered to have practice experience. Conversely, those who have never donated blood are classified as having no practice (10, 21, 27).

## Result

### Socio-demographic characteristics

The initial sample size for this study was 402 individuals; however, after the data collection process, a total of 393 students ultimately participated, resulting in an impressive response rate of 97.76%. This high response rate underscores the strong engagement and interest among the target population, which is critical for the reliability and validity of the study’s findings. Regarding the demographic composition of the participants reveals a diverse group. Among the respondents, 59% identified as male, while 41% identified as female. This gender distribution provides a balanced perspective on the knowledge levels regarding voluntary blood donation, allowing for a comprehensive analysis of potential differences in awareness and attitudes between genders (Table 1).

**Table 1:** Socio-demographic characteristics about voluntary blood donation among undergraduate regular student of WCU, Southcentral Ethiopia, 2023.

Sociodemographic characteristics		Number	Frequency
Gender	Male	232	59%
	Female	161	41%
	Total	393	100%
Age	18–22	142	36.1%
	23–28	172	43.8%
	28–34	63	16%
	>34	16	4.1%
Ethnic group	Hadiya	67	17%
	Kambata	23	65.9
	Gurage	27	6.9%
	Amhara	118	30%
	Oromo	103	26.2%
	Other	55	14%
College	Medicine and health science	132	33.6%
	Engineering	88	22.4%
	Agriculture	72	18.3%
	Business	101	25.7%
Study year	2nd	101	25.7%
	3rd	220	56%
	4th	28	7.1%
	5th	44	11.2%
Previous residence	Urban	145	36.9%
	Rural	248	63.1%
Marital status	Married	36	9.2
	Single	357	90.8%
Religion	Protestant	116	29.5%
	Muslim	76	19.3%
	Orthodox	172	43.8%
	Other	29	7.4%

In terms of religious affiliation, the participants represented a variety of faiths, with 43.8% identifying as Orthodox Christians, making it the largest religious group within the sample. Protestants comprised 29.5%, while Muslims accounted for 19.3% of the participants. Additionally, 7.4% identified with other faiths. This diversity in religious backgrounds may influence perceptions and attitudes toward blood donation, highlighting the need for culturally sensitive educational interventions. Geographically, the study also captured a significant demographic divide between urban and rural residents. A majority of participants, specifically 248 individuals (63.1%), resided in rural areas, while 145 participants (36.9%) hailed from urban settings. This distinction is particularly relevant, as it may affect access to healthcare resources, including blood donation facilities, and could inform targeted outreach strategies aimed at increasing voluntary blood donation rates in different communities (Table 1).

### Level of knowledge toward voluntary blood donation

The assessment of overall knowledge levels regarding voluntary blood donation was conducted by tallying the number of correct answers provided by each participant in the study. The analysis revealed that a significant majority, specifically 77.6% of respondents, scored at or above the 50^th^ percentile. This indicates that these individuals possess a good understanding of the subject matter related to voluntary blood donation. In contrast, 22.4% of the participants scored below the 50th percentile, which classified them as having poor knowledge about the topic (Tables 2, 3).

**Table 2:** Knowledge of undergraduate regular student of WCU on voluntary blood donation, WCU, Southcentral Ethiopia, 2023.

Knowledge related question			Number	Percentage
Had you heard/seen about blood donation before the study		Yes	338	86%
		No	55	14%
If your answer to Q. no 1 is “yes,” from where did you hear or see those messages (More than one answer is possible.)		Health profession	167	42.5%
		Print media	158	40.2%
		Electronic media	209	53.2%
		School/university	248	63.1%
Minimum criteria for donating blood	Age	<18	132	33.6%
		18–65	261	66.4%
	Weight	<45 kg	54	13.7%
		>45 kg	339	86.3%
	Good health	Yes	362	92.1%
		No	31	7.9%
Do you know the most common blood group types		Yes	171	43.5%
		No	222	56.5%
Do you know your own blood group type		Yes	89	22.6%
		No	304	77.4%
At what interval are blood donors able to donate		Once every 3 month	226	57.5%
		Other(once a week, month, 6 month)	167	42.5%
During blood donation, how much blood should be donated by a donor		<500 mL	257	65.4%
		>500 mL	136	34.6%
Can a person be infected by receiving blood transfusion		Yes	256	65.1%
		No	137	34.9%

**Table 3:** Level and distribution of knowledge level on voluntary blood donation among regular student of different college of WCU, Southcentral Ethiopia, 2023.

		College of student				Total (%)
		CMHS (%)	Engineering	Agriculture	Business and economics	
Knowledge level	Poor (%)	8 (9.09%)	25 (28.4%)	20 (22.72%)	35 (39.7%)	88 (22.4%)
	Good (%)	124 (40.6%)	63 (20.7%)	52 (17.04%)	66 (21.6%)	305 (77.6%)

Diving deeper into specific awareness levels, it was found that a noteworthy 86% of participants had heard of voluntary blood donation, suggesting a general familiarity with the concept. However, when it came to more detailed knowledge, the results varied. Approximately 66.4% of respondents were aware of the age requirements for individuals eligible to donate blood, indicating a moderate level of understanding regarding who can participate in such initiatives. Furthermore, an even higher percentage, 86.3%, demonstrated awareness of the weight requirements necessary for voluntary blood donation, highlighting a strong recognition of this critical criterion (Tables 2, 3). These findings underscore the importance of targeted educational efforts to enhance knowledge among those who may fall into the category of poor knowledge, as well as to reinforce existing awareness among those who are already informed. Overall, while a substantial portion of the population exhibits good knowledge about voluntary blood donation, there remains room for improvement, particularly in ensuring that all potential donors are fully informed about eligibility criteria and the significance of their contributions (Tables 2, 3).

### Attitude toward voluntary blood donation

Regarding the overall attitudes assessed in the study, it was found that 79.6% of respondents held a favorable view toward voluntary blood donation (VBD). Specifically, 84.7% of participants believed that voluntary blood donation is a commendable practice, and 88.3% considered it the best form of blood donation. However, it is noteworthy that 65.9% of respondents expressed an unwillingness to donate blood voluntarily in the future (Tables 4, 5).

**Table 4:** Attitude about voluntary blood donation among undergraduate regular students of WCU, Southcentral Ethiopia, 2023.

Attitude question		Number	Frequency
What do you think about voluntary blood donation?	Good	333	84.7%
	Not good	60	15.3%
Among different blood donation type is voluntary blood donation is the best?	Yes	347	88.3
	No	46	11.7
Can something harmful happen to a blood donor during or after Blood donation?	Yes	285	72.5%
	No	108	27.5%
What can happen to a blood donor during or after donation?	Contract infection	134	34.1%
	Feel sick	138	35.1%
	Temporary weakness	182	46.3%
Your donation will encourage others to donate	Disagree	126	32.1%
	Agree	267	67.9%
Willingness to donate blood in the future	Yes	134	34.1%
	No	259	65.9%

**Table 5:** Level and distribution of Attitude level on voluntary blood donation among regular student of different college of WCU, Southcentral Ethiopia, 2023.

		College of student				Total (%)
		CMHS (%)	Engineering	Agriculture	Business and economics	
Attitude level	Unfavorable (%)	7 (8.75)	23 (28.75)	16 (20)	34 (42.5)	80 (21.4%)
	Favorable (%)	125 (39.93)	65 (20.76)	56 (17.89)	67 (21.4)	313 (79.6%)

### Practice on voluntary blood donation

**Figure 1:**
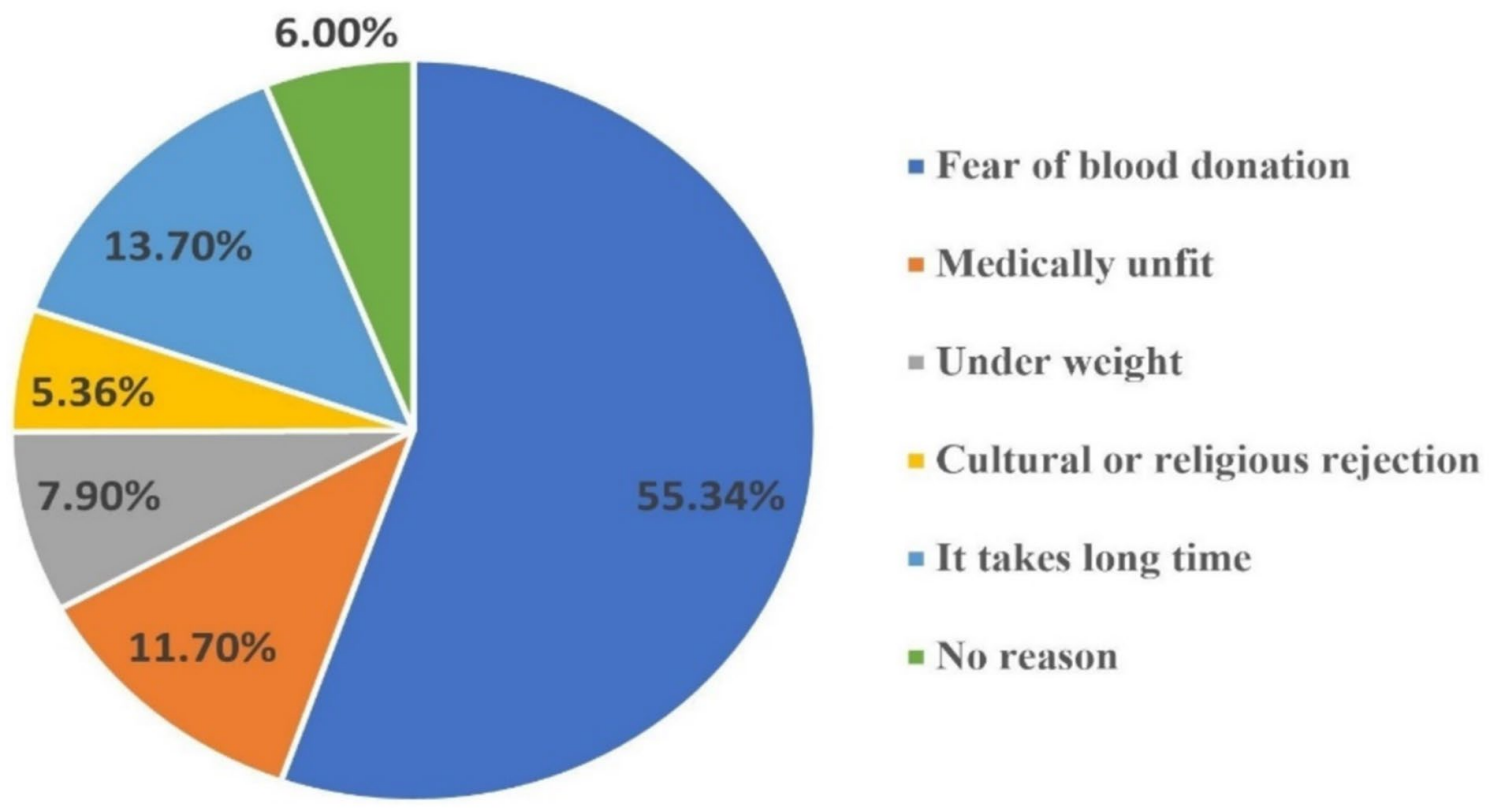
Reasons for not blood donation among undergraduate regular students of WCU, Southcentral Ethiopia, 2023.

The study found that fewer than a quarter of participants, specifically 76 individuals (19.3%), had ever donated blood at least once, while the remaining participants had never donated. Among those who had donated, 58 (76.3%) did so once, 11 (14.4%) donated twice, and 7 (9.2%) donated more than twice throughout their lifetime. Additionally, a significant majority (70.9%) of participants had not participated in any blood donation campaigns (Table 6; Figure 1).

**Table 6:** Practice and related information’s about voluntary blood donation among undergraduate regular students of WCU, Southcentral Ethiopia, 2023.

Practice related question		Number	Frequency
Have you ever donated blood before?	Yes	76	19.3%
	No	317	80.7%
If your answer to Q. no.1 is “Yes,” type of blood donation practiced	Replacement	4	1%
	Voluntary	72	18.3%
	Paid	0	0
If your answer to Q. no.1 is “Yes,” how many times do you donate?	Once	58	14.8%
	Twice	11	2.8%
	More than twice	56	1.8%
If your answer to Q. no.1 is “No,” what was the reason? (More than one answer is possible.)	Fear of blood donation	175	55.34%
	Medically unfit	37	11.7%
	Under weight	25	7.9%
	Cultural or religious rejection	17	5.36%
	It takes long time	44	13.7%
	No reason	19	6%
Have you taken blood donation campaigns?	Yes	117	29.1%
	No	285	70.9%

### Factors associated with practice toward voluntary blood donation among regular students of WCU, Southcentral Ethiopia, 2023

Variables with a *p*-value less than 0.25 in the bivariate analysis were selected for inclusion in the final multivariable logistic regression model to determine factors independently associated with voluntary blood donation. The multivariable analysis revealed that college, knowledge, and residence were significantly linked to blood donation practices. After adjusting for other independent variables. Students in the College of Medicine and Health Sciences were 2.53 times more likely to volunteer for blood donation than those in the College of Business and Economics (AOR: 2.53; 95% CI: (1.19–5.39)). Participants with good knowledge were 3.23 times more likely to donate blood voluntarily compared to those with poor knowledge (AOR: 3.23; 95% CI: (1.07–9.78)). Furthermore, individuals from urban areas were 2.27 times more likely to donate blood voluntarily than those from rural areas (AOR: 2.27; 95% CI: (1.31–3.94)) (Table 7).

**Table 7:** Factors associated with practice toward voluntary blood donation among undergraduate regular students of WCU, Southcentral Ethiopia, 2023.

Characteristics		practice level		Bivariate analysis	Multivariable analysis	
		Practice (%)	Not practice (%)	COR (95% CI)	AOR (95% CI)	*p*-value
Sex	Male	52(68.4)	180(56.8)	1		
	Female	24 (31.4)	137(43.2)	0.52(0.33–0.82)		
Age	18–22	21 (27.6)	121(38.2)	1		
	23–28	36 (47.4)	136(42.9)	1.53 (0.84–2.76)		
	29–34	13(17.1)	50(15.8)	1.49 (0.69–3.22)		
	>35	6(7.9)	10(3.2)	3.46(1.14–10.52)		
Religion	Protestant	29 (65.1)	89 (72.3)	1	1	
	Orthodox	38 (25.7)	184(23.5)	0.87 (0.50–1.51)	0.92 (0.51–1.68)	0.792
	Muslim	5(7.3)	71(2.7)	0.22 (0.08–0.59)	0.04 (0.08–0.69)	0.088^*^
	Other	4(7.3)	23(1.5)	0.53 (0.17–1.67)	0.45 (0.13–1.49)	0.189
College	Business and economics	12 (15.8)	89(28.1)	**1**	1	
	Engineering	7 (9.2)	81(25.6)	0.64 (0.24–1.71)	0.51 (0.19–1.40)	0.192
	Agriculture	9 (11.8)	63 (19.9)	1 (0.42–2.67)	0.88 (0.34–2.28)	0.786
	Medicine and health science	48(63.2)	84 (26.5)	4.24 (2.11–8.53)	2.53 (1.19–5.39)	0.016^∗^
Year of study	2nd	13 (17.1)	88 (27.8)	1		
	3rd	53 (69.7)	167 (52.7)	2.15 (1.11–4.15)		
	4th	7 (9.2)	21 (6.6)	2.26 (0.80–6.35)		
	5th	3 (3.9)	41 (12.9)	0.49 (0.13–1.83)		
Previous residence	Rural	34 (44.7)	214 (67.5)	1		
	Urban	42 (55.3)	103 (32.5)	2.57 (1.54–4.27)	2.27 (1.31–3.94)	0.004^*^
Knowledge status	Poor	4 (5.3)	84 (26.5)	1	1	
	Good	72 (94.7)	233 (73.5)	6.49 (2.30–18.31)	3.23 (1.07–9.78)	0.038^*^
Attitude status	Not favorable	7 (9.2)	73 (33)	1		
	Favorable	69 (90.8)	244 (77)	2.95 (1.29–6.69)		

Where: COR, Crude odds ratio; AOR, Adjusted odds ratio; CI, Confidence interval; ^**∗**^statically significant.

## Discussion

Globally, the shortage of blood supply has escalated into a critical public health issue, primarily driven by the increasing demand for blood due to advancements in medical procedures and rising rates of chronic illnesses that require blood transfusions (3, 4). This challenge is particularly pronounced in developing countries, where healthcare systems are often under-resourced and lack the necessary infrastructure to support consistent blood donation efforts (15, 28). The growing demand for blood, coupled with inadequate supply, poses significant challenges for health planners and policymakers who are tasked with ensuring that safe and sufficient blood is available for patients in need (29).

To effectively address the blood supply shortage, it is essential to comprehend the knowledge, attitudes, and practices (KAP) surrounding voluntary blood donation (VBD). A nuanced understanding of KAP can inform targeted strategies to enhance blood donation rates. By identifying gaps in knowledge and addressing misconceptions, health authorities can implement educational campaigns that promote the importance of blood donation, thereby fostering a culture of giving within communities. This study aimed to assess KAP levels related to VBD among regular students at Wachemo University in Hosanna, Central Ethiopia, in 2023. University students represent a vital demographic for potential blood donors due to their age, health status, and social engagement. By focusing on this group, the study seeks to provide insights that can help improve voluntary blood donation rates within this critical age bracket.

The findings of this study reveal critical insights into the KAP surrounding voluntary blood donation among undergraduate students at Wachemo University. About 77.6% of respondents demonstrated good knowledge about voluntary blood donation. While this is a commendable level of awareness, it is comparable with study reported in Arsi University (79.4%) (21) and lower than the results from studies conducted in Malaysia (97.1%) (30), Addis Ababa University (AAU) (83.7%) (31). These differences may reflect variations in educational outreach, cultural attitudes toward blood donation, and access to information about its importance. Conversely, our results were higher than those from studies in Nepal (32.4%) (32), Kerala (35%) (33), and Ambo University (67.6%) (19), indicating that while some regions have made strides in promoting VBD knowledge, others still face significant challenges.

A significant portion of respondents demonstrated adequate knowledge about blood donation; however, gaps remain, particularly regarding misconceptions about the donation process and eligibility criteria. Notably, 65.4% of participants were aware of the volume of blood typically donated during a session, and 86.3% knew the minimum weight requirement for donors. This aligns with previous studies indicating that knowledge deficits are prevalent among young adults (19). However, these figures are lower than those reported among health science students at Addis Ababa University (91.4 and 90.4%, respectively) (31). Educational interventions focusing on dispelling myths and providing clear information about the donation process could enhance knowledge levels and subsequently improve attitudes.

Attitudes toward blood donation were generally positive among participants, with many recognizing its importance for community health. This study result found that 79.6% of respondents held a favorable disposition toward donating blood. This percentage surpasses findings from studies in Iraq (68.7%) (34), India (57.8%) (35), and AAU (68%) (31). However, only 34.1% expressed a willingness to donate blood in the future, which is significantly lower than the 85.5% reported in a study at the University of Gondar (20). Additionally, some students expressed concerns about potential health risks and personal discomfort associated with donating, which highlighted that fear and misinformation significantly influence attitudes toward blood donation among young adults. Addressing these fears through targeted educational campaigns that emphasize the safety and benefits of donating could help mitigate negative perceptions.

In terms of actual practice, the study found a notable discrepancy between students’ willingness to donate and their actual participation rates. Interestingly, only 19.3% of respondents reported having donated blood at least once. This figure is notably lower than findings from studies in India (47.5%) (36), Malaysia (29.7%) (30), Namibia (28%) (37), and Nigeria (35.4%) (38). However, it is higher than the 12.5% reported among graduating health science students at Gondar University. The low rates of actual donation could be influenced by various socio-cultural factors, including misconceptions about blood donation, fear of needles or medical procedures, and a lack of organized blood donation campaigns within the university environment (39).

In this study, common reasons cited for not donating included fear of the donation process (55.34%), time constraint (13.7%) and concerns about being medically unfit (11.7%). These findings align with previous research indicating that fears and misconceptions significantly hinder individuals from participating in blood donation initiatives (19, 21, 40). To bridge this gap, organizing on-campus blood donation drives and providing flexible scheduling options could facilitate higher participation rates. Moreover, cultural factors also play a significant role in shaping KAP regarding blood donation.

In some communities, cultural beliefs may discourage individuals from donating blood due to fears surrounding health implications or stigma associated with the act. Engaging community leaders and influencers can help address these cultural barriers and promote a positive narrative around voluntary blood donation.

Multivariate analysis revealed that factors such as college affiliation, previous residence, and knowledge were significantly associated with VBD practice among respondents. This finding contrasts with studies conducted in Tanzania and Samara University, where knowledge and college affiliation did not show a correlation with donation practices (10, 41). The significance of college affiliation suggests that students from certain colleges may have more robust exposure to blood donation campaigns or educational initiatives. This could be due to differences in curriculum focus, extracurricular activities, or partnerships with health organizations that promote blood donation. Colleges with active health programs may foster a more positive attitude toward VBD, thereby encouraging students to engage in donation practices.

The impact of previous residence indicates that students’ backgrounds may play a crucial role in shaping their attitudes and practices regarding blood donation. Students who come from regions with established blood donation programs or cultural norms favoring donation may be more likely to participate in VBD. Conversely, those from areas where blood donation is less emphasized might carry misconceptions or lack motivation to donate. The strong association between knowledge and VBD practice underscores the importance of education in dispelling myths and enhancing understanding about the benefits and safety of blood donation. Increased awareness can lead to greater willingness to donate, as individuals become informed about the critical need for blood and the positive impact their contributions can have on community health. In this study, age, gender, and religion were not found to be associated with VBD practice—an outcome that may reflect specific socio-demographic dynamics within the university population.

The findings of this study highlight the necessity for a comprehensive strategy that includes educational campaigns aimed at raising awareness about the significance and advantages of voluntary blood donation, as well as encouraging actual participation. In addition, integrating topics related to blood donation and its importance into the university curriculum can help cultivate a culture of generosity among students. This could involve workshops, seminars, or guest lectures featuring health professionals. Furthermore, encourage partnerships between universities and local health authorities to facilitate regular blood donation drives on campus. This would make it more accessible for students to donate blood and could help increase overall donation rates.

### Limitations of the study

Although the study offers valuable insights into the knowledge, attitudes, and practices related to voluntary blood donation among Wachemo University students, its cross-sectional design restricts the ability to determine causal relationships and monitor changes over time. Another notable limitation in the current study is the lack of consideration for design effects during sample size calculation. Future research would benefit from employing longitudinal designs and more robust sample size methodologies to deepen the understanding of KAP dynamics in this area.

## Conclusion and recommendations

The findings reveal that although 77.6% of students have a solid understanding and 79.6% hold positive views on blood donation, only 19.3% participate in donating blood. To boost participation, the institution should collaborate with key stakeholders to address barriers to voluntary blood donation. Furthermore, incorporating blood donation topics into the curriculum could foster a culture of generosity, essential for raising blood donation rates at the university and beyond, ensuring sufficient blood supplies for patients in need of transfusions.

## Data Availability

The original contributions presented in the study are included in the article/supplementary material, further inquiries can be directed to the corresponding author.

## References

[1] Fong IW. Blood Transfusion-Associated Infections in the Twenty-First Century: New Challenges. Cur-rent Trends and Concerns in Infectious Diseases. (2020) 7:191–215.

[2] Napolitano LM Kurek S Luchette FA Corwin HL Barie PS Tisherman SA. Clinical practice guideline: red blood cell transfusion in adult trauma and critical care. Crit Care Med. (2009) 37:3124–57. doi: 10.1097/CCM.0b013e3181b39f1b, PMID: 19773646

[3] World Health Organization. Towards 100% Voluntary Blood Donation: A Global Framework for Action. Geneva: World Health Organization. (2010). 2, Voluntary blood donation: foundation of a safe and sufficient blood supply”. Available from: https://www.ncbi.nlm.nih.gov/books/NBK305666 26225399

[4] Jenny HE Saluja S Sood R Raykar N Kataria R Tongaonkar R. Access to safe blood in low-income and middle-income countries: lessons from India. BMJ Glob Health. (2017) 2:e000167. doi: 10.1136/bmjgh-2016-000167, PMID: 30206488 PMC5584485

[5] Ahmed A. Knowledge, attitude, practice and associated factors of voluntary blood donation among undergraduate students in Hargeisa University. J Community Med Health Edu. (2017) 7:547–59. doi: 10.4172/2161-0711.1000547

[6] Myers DJ Collins RA. Blood donation. Treasure. Island (FL): StatPearls Publishing (2023). 30247842

[7] Mulatu K Hailu T Yegezu A Tena B. Assessment of knowledge, attitude and practice on blood donation in Aman Sub City residents, south west, Ethiopia, 2015. Health Sci J. (2017) 11:485. doi: 10.21767/1791-809X.1000485

[8] Tsega A Mullualem D Tadesse BA. Assessment of knowledge, attitude, practice, and associated factors of voluntary Blood donation in selected towns of Awi zone, Injibara, Ethiopia. Biomed Res Int. (2024) 2024:6069684. doi: 10.1155/2024/6069684, PMID: 39376255 PMC11458273

[9] Enawgaw B Yalew A Shiferaw E. Blood donors’ knowledge and attitude towards blood donation at North Gondar district blood bank, Northwest Ethiopia: a cross-sectional study. BMC Res Notes. (2019) 12:1–6. doi: 10.1186/s13104-019-4776-0 31694710 PMC6836355

[10] Tadesse W Ayalew Y Yisma E Liben ML Wudu M. Knowledge, attitude, practice and associated factors towards voluntary blood donation among regular health science students of Samara University, Ethiopia. Health Sci J. (2018) 12:542. doi: 10.21767/1791-809X.1000542

[11] Ahmed Z Zafar M Khan AA Anjum MU Siddiqui MA. Knowledge, attitude and practices about blood donation among undergraduate medical students in Karachi. J Infect Dis Therap. (2014) 2, 1–5. doi: 10.4172/2332-0877.1000134

[12] FMoH E. Health sector transformation plan. Addis Ababa: Addis Ababa Ethiopia (2015).

[13] Melesse DY Tadele A Mulu S Spicer N Tadelle T Wado YD. Learning from Ethiopia’s success in reducing maternal and neonatal mortality through a health systems lens. BMJ Glob Health. (2024) 9:e011911. doi: 10.1136/bmjgh-2023-011911 PMC11085893 38770809

[14] Tesfaye G Loxton D Chojenta C Assefa N Smith R. Magnitude, trends and causes of maternal mortality among reproductive aged women in Kersa health and demographic surveillance system, eastern Ethiopia. BMC Womens Health. (2018) 18:198. doi: 10.1186/s12905-018-0690-1, PMID: 30518368 PMC6282369

[15] Bates I Chapotera GK McKew S van den Broek N. Maternal mortality in sub-Saharan Africa: the contribution of ineffective blood transfusion services. BJOG. (2008) 115:1331–9. doi: 10.1111/j.1471-0528.2008.01866.x, PMID: 18823485

[16] Mecha A Erchafo B. Blood donation intentions and predictors among hosanna town dwellers, south nation nationality peoples region, Ethiopia. J Family Med Prim Care. (2022) 11:5320–6. doi: 10.4103/jfmpc.jfmpc_1287_21, PMID: 36505538 PMC9731061

[17] Zeleke AM Azene ZN. Willingness and its associated factors for blood donation in Gondar town, Northwest Ethiopia: a community-based cross-sectional study. Hygiene. (2022) 2:212–25. doi: 10.3390/hygiene2040019

[18] Darega B Dida N Tesfaye T Lencha B. Voluntary blood donation practices and associated factors among regular undergraduate Madawalabu University students, Southeast Ethiopia: a facility-based cross sectional study. J Blood Disord Transfus S. (2015) s5:S5–S005. doi: 10.4172/2155-9864.1000S5-005

[19] Nigatu A Demissie DB. Knowledge, attitude and practice on voluntary blood donation and associated factors among ambo university regular students, Ambo Town, Ethiopia. J Commun Med Health Educ. (2014):4. doi: 10.4172/2161-0711.1000315

[20] Melku M Asrie F Shiferaw E Woldu B Yihunew Y Asmelash D. Knowledge, attitude and practice regarding blood donation among graduating undergraduate health science students at the University of Gondar, Northwest Ethiopia. Ethiop J Health Sci. (2018) 28:571–82. doi: 10.4314/ejhs.v28i5.8 30607072 PMC6308782

[21] Gebresilase HW Fite RO Abeya SG. Knowledge, attitude and practice of students towards blood donation in Arsi university and Adama science and technology university: a comparative cross sectional study. BMC Hematol. (2017) 17:17. doi: 10.1186/s12878-017-0092-x 29201379 PMC5697394

[22] Ogundeji SP Ajayi OD Busari OE Ogundeji OA Adepoju OA Esan FG. Knowledge, attitude, and perception towards voluntary blood donation among university students in Nigeria. ISBT Sci Ser. (2021) 16:85–91. doi: 10.1111/voxs.12614

[23] Beyene GA. Voluntary Blood donation knowledge, attitudes, and practices in Central Ethiopia. Int J Gen Med. (2020) 13:67–76. doi: 10.2147/IJGM.S246138, PMID: 32184648 PMC7061424

[24] Eltewacy NK Ali HT Owais TA Alkanj S Elbahnasawy M Ahmed AHB. Unveiling blood donation knowledge, attitude, and practices among 12,606 university students: a cross-sectional study across 16 countries. Sci Rep. (2024) 14:8219. doi: 10.1038/s41598-024-58284-4, PMID: 38589387 PMC11001850

[25] Ibrahim AA Koç M. Knowledge level, motivators and barriers of Blood donation among students at Qatar University (2021) 9:926. doi: 10.3390/healthcare9080926 PMC8391523 34442063

[26] Cochran WG. Sampling Techniques. New York: Wiley (1977).

[27] Mussema A Bawore SG Abebaw T Tadese W Belayineh M Yirga A. Voluntary blood donation knowledge, attitude, and practice among adult populations of hosanna town, South Ethiopia: a community-based cross-sectional study. Front Public Health. (2023) 11:1141544. doi: 10.3389/fpubh.2023.1141544, PMID: 37383266 PMC10296759

[28] Mpimbaza A. Blood transfusion in sub-Saharan Africa: walking a tightrope. Lancet Haematol. (2020) 7:e774–5. doi: 10.1016/S2352-3026(20)30329-X, PMID: 33091345

[29] Goyal P Kumar A Khan H Kumar R. Effect of educational intervention on awareness and attitude regarding voluntary Blood donation among rural people: a quasi-experimental study. Indian J Community Health. (2023) 35:526–8. doi: 10.47203/IJCH.2023.v35i04.021

[30] Elnajeh M Ghazi HF Abdalqader MA Baobaid MF. Knowledge, attitude and practice towards blood donation and its associated factors among university students in Shah Alam, Malaysia. Int J Commun Med Public Health. (2017) 4:2230–3. doi: 10.18203/2394-6040.ijcmph20172811

[31] Tesfaye M Misganaw C Tenkir M Deresea A Tessema TT Taye H. The level and associated factors of knowledge, attitude and practice of Blood donation among health science students of Addis Ababa university. Int J Med Health Sci. (2014) 1:105–18.

[32] Singh S Rijal TD Katwal B. Blood Donor’s knowledge, attitude and practice towards Blood donation among voluntary Blood donors at Nepal red cross society central Blood transfusion service Centre. J Nepalgunj Med College. (2023) 21:5–9. doi: 10.3126/jngmc.v21i2.62714

[33] Aslami A Jobby A Simon S Nazarudeen N Raj P Ramees M. Assessment of knowledge, attitude and practice (kap) of blood donation among mbbs students of a medical college in Kollam, Kerala. J Evol Med Dent Sci. (2015) 4:6086–95. doi: 10.14260/jemds/2015/887

[34] Devi VK Thirumurugan E Karthick R Indian EA Abinaya G Hemalatha R. Assessment of knowledge, attitude, and practice of blood donation among paramedical students of a medical college in Chennai, Tamil Nadu. Iraqi J Hematol. (2024) 13:71–8. doi: 10.4103/ijh.ijh_21_24

[35] Govindasamy V Sivasankaran D Purushothaman V. Knowledge, attitude and practice regarding blood donation among medical students of Tamil Nadu- a cross sectional study. Int J Commun Med Public Health. (2019) 6:4583. doi: 10.18203/2394-6040.ijcmph20194532

[36] Giri P Phalke DB. Knowledge and attitude about Blood donation amongst undergraduate students of Pravara Institute of Medical Sciences Deemed University of Central India. Annals Tropic Med Public Health. (2012) 5:569. doi: 10.4103/1755-6783.109274

[37] Ojulong J Muhoza JCH Monteiro L. Barriers that prevent health science students from donating blood in an African setting. Int J Commun Med Public Health. (2016) 3:3544–7. doi: 10.18203/2394-6040.ijcmph20164289

[38] Ugwu NI Ugwu CN Oti W Uneke CJ. Pattern of blood donation practices among students of a Nigerian University. Int Blood Res Rev. (2019) 26:1–8. doi: 10.9734/ibrr/2019/v9i330102

[39] Zanin TZ Hersey DP Cone DC Agrawal P. Tapping into a vital resource: understanding the motivators and barriers to blood donation in sub-Saharan Africa. African J Emerg Med. (2016) 6:70–9. doi: 10.1016/j.afjem.2016.02.003, PMID: 30456070 PMC6233251

[40] Kagoya C Gavamukulya Y Jonah SD. Knowledge, perceptions and practices towards blood donation among undergraduate medical students in an upcountry Ugandan university: a mixed methods study. Glob Public Health. (2024) 19:2311679. doi: 10.1080/17441692.2024.2311679, PMID: 38325404

[41] Florian FF. Barriers and drivers of voluntary blood donation in Katavi and Kilimanjaro regions: A comparative cross-sectional study of two regions with high and moderate blood collection rates in. Tanzania: NM-AIST (2023).

